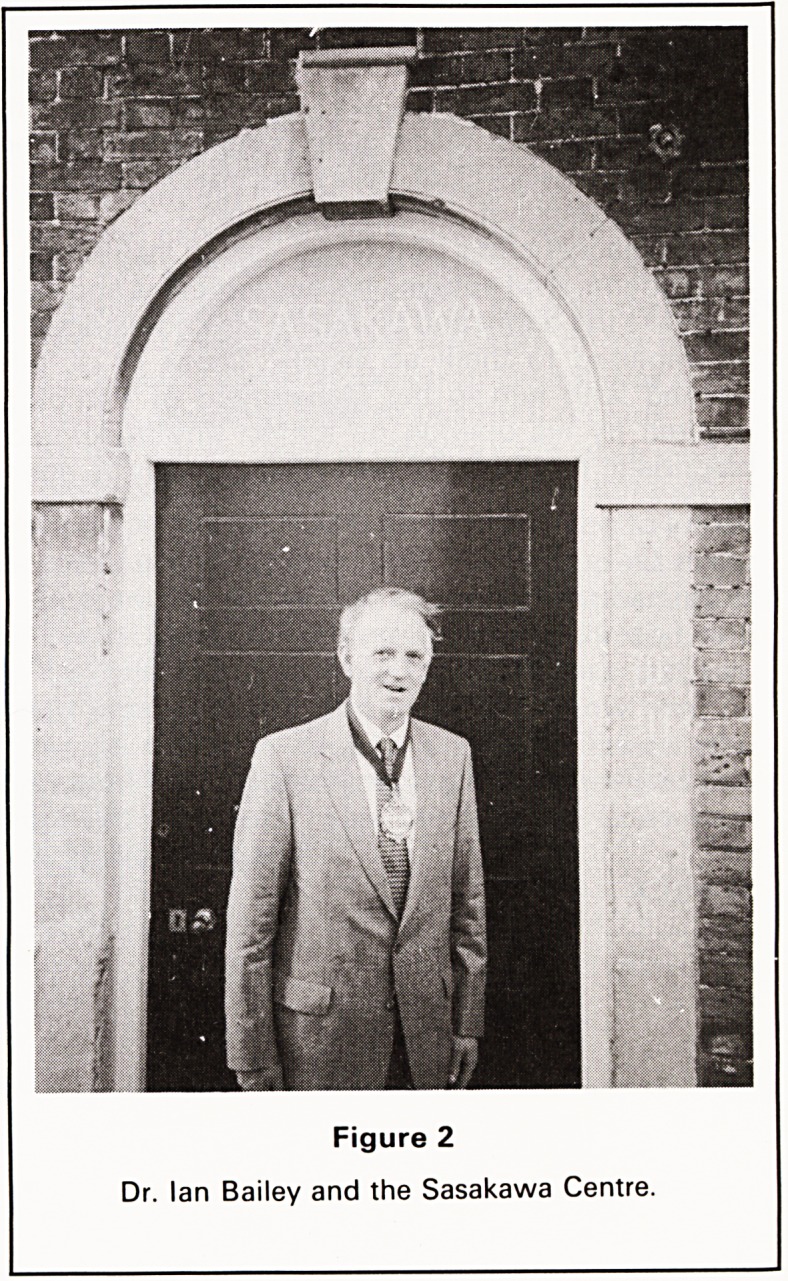# Bristol Medico—Chirurgical Society Summer Meeting

**Published:** 1985-07

**Authors:** 


### Bristol Medico-Chirurgical Society's Summer Meeting

Held at the Jenner Museum, The Chantry, Berkeley, Gloucestershire

Wednesday, 8th May 1985

This was the final meeting in the year of Dr. Ian Bailey's Presidency and was fittingly held in the new Jenner Museum at Berkeley shortly after the official opening by Mr. Sasakawa, the millionaire Japanese industrialist whose gift to the Jenner Trust of half a million pounds enabled the transformation to take place. The original Jenner Museum was set up in 1965 by the Jenner Trust stimulated by the late Dr. Malcolm Campbell, in the cottage at Berkeley that Jenner built for James Phipps, the first person he vaccinated. Jenner's original home in Berkeley was the fine 18th century house, The Chantry, which in

**Figure 1**

Dr. and Mrs. Bailey ready to welcome members to the new Jenner Museum.

**Figure 2**

Dr. Ian Bailey and the Sasakawa Centre.

1885 became the Vicarage. When it became known that the Church Authorities wished to dispose of The Chantry, a joint appeal was made by the Jenner Trust and the British Society of Immunology to raise funds to purchase The Chantry, transfer the museum to it and establish a small conference centre. It was thanks largely to the gift from Mr. Sasakawa that this became possible. Mr. Sasakawa is Chairman of the Japanese Shipbuilding Federation and his charitable donations have been enormous. He financed the W.H.O. Smallpox Campaign, which led to the virtual elimination of this disease world-wide and has recently given large donations to the leprosy campaign. He founded a Chair of Japanese Studies at Oxford University and donated a nine million pound fund to foster good relations between the U.K. and Japan. The Chantry has been renovated at a cost of £200,000 and the large building adjacent which, in Jenner's time, was used as a stable, has been turned into a conference centre.

We were lucky with the weather for, in a month when the skies had been mostly grey, the sun shone for us and the President stood outside the door of The Chantry, wearing his chain of office, to welcome members of the Society. We had the opportunity to see the new Museum and then went over to the Conference Centre for the meeting. Dr. Bailey introduced our Speaker, Canon Gethin Jones, who delivered his address 'The Chantry and its History'. Nobody could be better placed to speak on this subject for, as the last Vicar of Berkeley but one, it had been his home for many years. It was during this time that he developed his interest in Jenner which stimulated the researches which have made him a leading authority on the history of the Jenner family. He spoke with his customary animation and enthusiasm and his talk was much enjoyed and warmly applauded. We then repaired to the Berkeley Arms for an excellent salmon buffet supper in the large upstairs dining room. After dinner our President introduced Professor Perry, who is Chairman of the Jenner Trust, and asked him to speak on Jenner. This in a sense he declined to do by telling us that we all knew all about Jenner and had heard enough about him today anyway. Nevertheless he managed to find things to say about Jenner that many of us, including your correspondent, had not heard before, including a poetic gem 'Signs of Rain' written by Jenner, humorous and full of country lore. This delightful verse, tellingly recited from memory, made the perfect end to a memorable evening.

## Figures and Tables

**Figure 1 f1:**
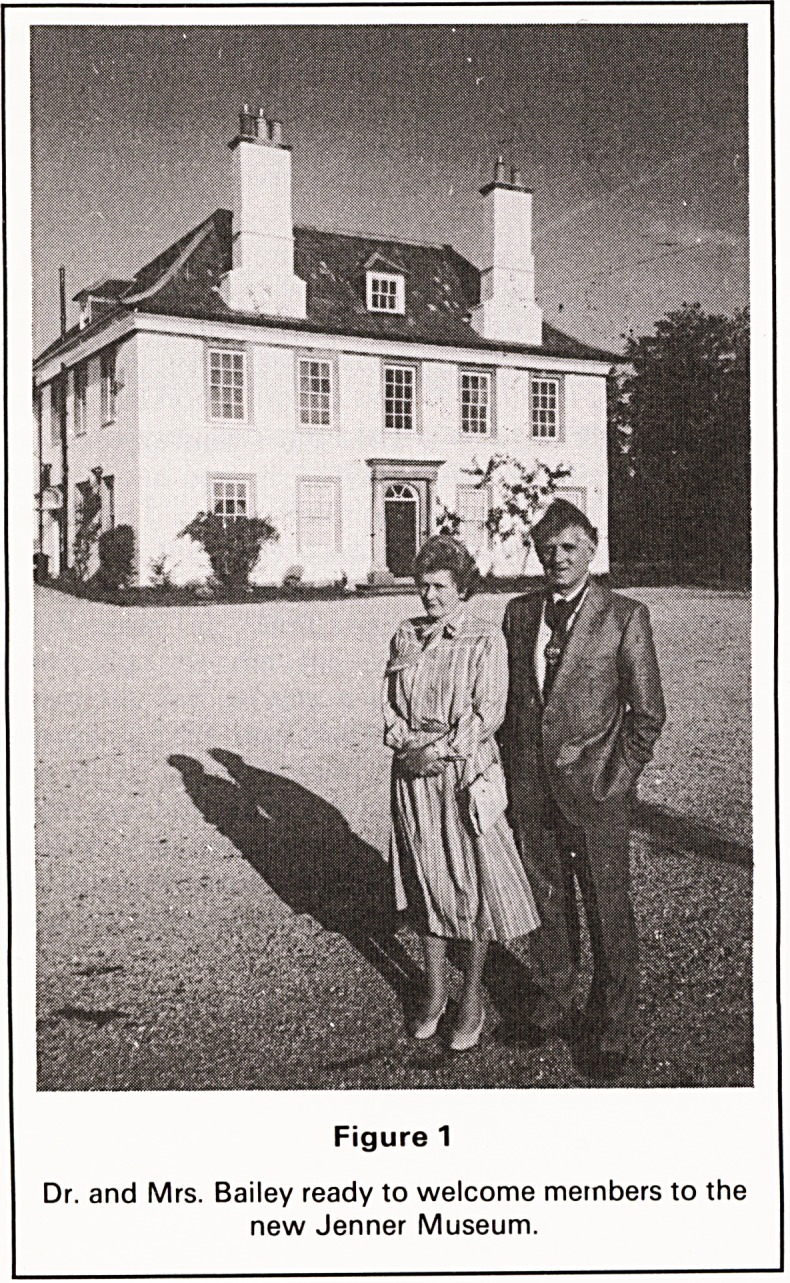


**Figure 2 f2:**